# Failure of cervical arthroplasty in a patient with adjacent segment disease associated with Klippel-Feil syndrome

**DOI:** 10.4103/0019-5413.77139

**Published:** 2011

**Authors:** Ioannis D Papanastassiou, Ali A Baaj, Elias Dakwar, Mohammad Eleraky, Frank D Vrionis

**Affiliations:** H. Lee Moffitt Cancer Center and Research Institute, Neurooncology Program, University of South Florida College of Medicine, 12902 Magnolia Drive, Tampa, Florida, 33612, USA

**Keywords:** Adjacent segment disease, cervical arthroplasty, Klippel-Feil

## Abstract

Cervical arthroplasty may be justified in patients with Klippel-Feil syndrome (KFS) in order to preserve cervical motion. The aim of this paper is to report an arthroplasty failure in a patient with KFS. A 36-year-old woman with KFS underwent two-level arthroplasty for adjacent segment disc degeneration. Anterior migration of the cranial prosthesis was encountered 5 months postoperatively and was successfully revised with anterior cervical fusion. Cervical arthroplasty in an extensively stiff and fused neck is challenging and may lead to catastrophic failure. Although motion preservation is desirable in KFS, the special biomechanical features may hinder arthroplasty. Fusion or hybrid constructs may represent more reasonable options, especially when multiple fused segments are present.

## INTRODUCTION

Adjacent segment disease (ASD) represents the pathological degeneration of the intervertebral discs, observed at the level(s) adjacent to a previously fused motion segment. Whether arthrodesis accelerates the development of ASD, or this observed degenerative process is merely a part of the natural history of spondylosis is a heavily contested proposition.

Cervical disc arthroplasty devices have been developed as an alternative to fusion techniques, with the aim of preserving motion and diminishing the rate of ASD acceleration.^1^ However, little is known as to whether Total Disc Replacement (TDR) is also effective in the treatment of the degenerated disc adjacent to a prior fusion, especially in patients with Klippel-Feil syndrome (KFS) who have “cod-fish” narrow vertebrae with resulting small endplates.

We present a patient with KFS who developed ASD above and below the autofused segments. These adjacent segments were treated with TDR. To the best of our knowledge, there have been two previous reports of successful cervical disc arthroplasty to treat adjacent segment disease in KFS. However, in this patient, we encountered failure in one of the inserted prosthesis.

## CASE REPORT

**Figure 1:**
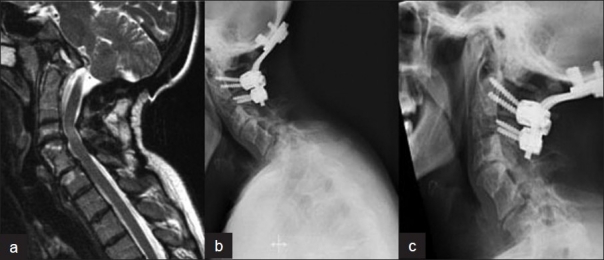
Mid sagittal T2WI (a) of cervical spine shows autofusion C5-6 with disc degeneration at C4-5 and C6-7. Lateral X-rays of cervical spine, flexion (b) extension (c) views show occipito cervical fusion performed before and some residual motion in the nonfused segment

**Figure 2:**
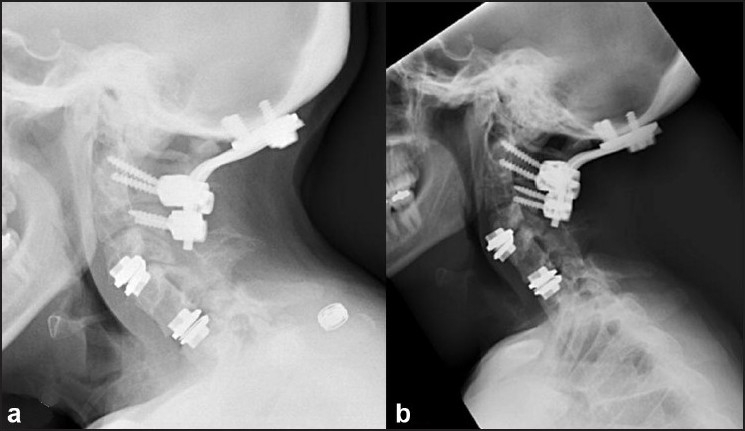
Immediate postoperative lateral radiograph (a) demonstrating two-level arthroplasty. 5 months follow-up lateral radiograph (b) shows extrusion and anterior migration of the C4-5 prosthesis

A 36-year-old woman having KFS presented with symptoms of axial neck pain radiating to the shoulders bilaterally, refractory to standard conservative therapy. She had previously undergone an occiput-C3 posterior fusion for atlanto-occipital instability and dysphagia [Figure 1a]. The C5/6 segment was congenitally autofused secondary to her KFS. Magnetic resonance imaging of the cervical spine demonstrated disc degeneration above and below the congenitally fused segment [Figure 1b,c]. Flexion/extension views showed some residual motion in the non-fused segments. Cervical arthroplasty (*ProDisc*-C, size 5 × 12 × 14 mm, Synthes, Inc, West Chester, PA) was performed at the C4/5 and C6/7 segments using the standard anterior cervical approach. Discectomy and resection of the posterior longitudinal ligament were carried out at both levels until the neural foramen were well decompressed. Although the procedure was uneventful, it was more technically demanding secondary to her short neck, especially at the cranial level. Immediate postoperative radiographs showed satisfactory placement of the implants [Figure 2a].

**Figure 3:**
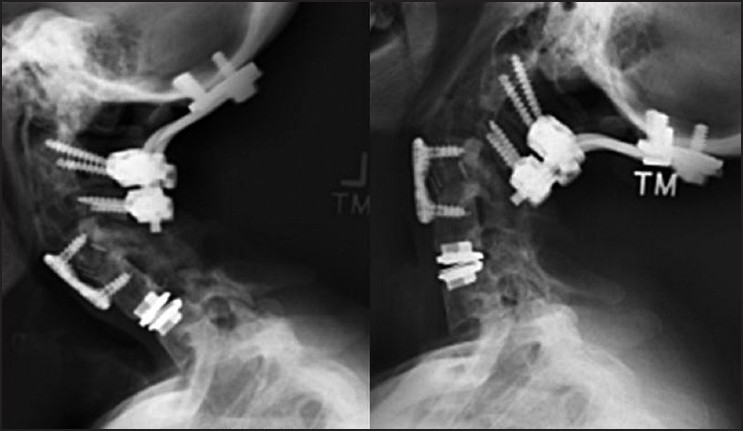
Final flexion/extension radiographs after prosthesis revision and anterior cervical fusion. Motion is retained at the level of the distal artificial disc, 1 year after implantation

The patient’s postoperative course was complicated, with dysphagia and hoarseness reflecting probable irritation to the recurrent laryngeal nerve. This required placement of a temporary percutaneous gastrostomy tube. One month after surgery, she regained baseline swallowing and was pain free. However, approximately 5 months postoperatively, she began to re-experience difficulty swallowing. Repeat imaging studies demonstrated migration and extrusion of the cranial implant at the C4/5 level of more than 50% [Figure 2b]. She was re-admitted for revision surgery of the cervical arthroplasty device. Despite the desire to preserve motion and retain the arthroplasty, due to heterotopic ossification (HO) in the posterior third of the disc space and the poor bone quality in the inferior endplate, an intraoperative decision was made to proceed with anterior cervical fusion. Her postoperative course was uneventful and she is clinically symptom free at the 6-month follow-up after the revision surgery. In addition, the flexion/extension films obtained 1 year after the disc replacement demonstrated motion at the existing artificial disc [Figure 3].

## DISCUSSION

Selection of the appropriate treatment strategy for symptomatic intervertebral disc degeneration adjacent to a previously fused segment is controversial. Historically, the treatment of choice has been to excise the disc and extend the fusion. Given the biomechanical and clinical evidence suggesting possible acceleration of ASD after fusion, the role of motion-preserving devices in cervical spondylosis has gained wide interest.^2^–^4^

The incidence of radiographic adjacent level changes has been reported to be 3.5 times higher in patients who underwent cervical arthrodesis when compared to arthroplasty.^5^ Robertson **et al**. also reported that the incidence of symptomatic adjacent level disease was statistically greater in patients treated with fusion when compared to those treated with the artificial disc.^4^

Cervical arthroplasty has been considered a safe operation, with most complications related with the anterior approach (dysphagia, neurovascular/visceral injury, dural tears). Technical pitfalls or patient selection may certainly compromise the final outcome. Longer-term complications include loss of mobility, failure or loosening of the implant.^6^,^7^ To the best of our knowledge, anterior migration of the device has been rarely reported for the *Bryan* cervical disc (Medtronic Inc, Memphis TN)^7^,^8^ but not for the *ProDisc*-C. HO, as seen in our case, frequently occurs and can be as high as 71%.^9^

Klippel-Feil syndrome is a congenital disorder highlighted by vertebral column and skeletal anomalies, deafness, genitourinary and cardiovascular abnormalities.^10^ Short neck, autofused spinal segments, neurovascular variability and atlanto-occipital instability are only a few examples of why cervical spine disease represents a surgical challenge in patients with KFS.^11^–^13^ Various classification systems have been proposed.^14^–^15^ According to Samartzis^14^ classification, our patient would fit into type II category, which is characterised by non-contiguous fused segments and intermediate symptoms severity. Furthermore, the decision to perform an arthrodesis or arthroplasty is difficult, as the natural history of KFS is poorly understood. There are two other published reports demonstrating the safety and effectiveness of TDR in KFS. Leung **et al**.^16^ implanted the Bryan artificial disc at C4/5 level and good short-term results were encountered. In another case report, Yi **et al**.^17^ performed a C6-7 arthroplasty and preservation of motion was maintained at 2 years after surgery. In a series of multilevel disc replacement, including a patient with congenital block vertebrae, somehow analogous to KPS, the authors report favourable results.^18^

In our patient with a congenitally fused C5-C6 level and Occipital-Cervical prior fusion, we performed an arthroplasty to preserve her remaining cervical range of motion (ROM) and potentially decrease the chance of ASD. However, this led to hardware failure and required revision with arthrodesis. Although the remaining disc showed satisfactory motion at 1 year, it is difficult to claim that this represents a clinical benefit in the context of the second operation. What may have been more beneficial is the use of a hybrid construct, especially in a patient with type II syndrome, although the literature data are insufficient to support yet such a notion. In a biomechanical study, Cho **et al**. showed that the hybrid group had no difference in ROM with the intact group, including adjacent segments, whereas the double fusion group had less motion and the double arthroplasty group increased ROM.^19^ In a clinical series of 24 patients with hybrid constructs, the authors demonstrated good ROM in the arthroplasty level with good clinical outcome and no complications, except for 2 cases of HO. They recommend doing the arthroplasty in the more mobile and less degenerative segment.^20^ The kinematics of segments adjacent to fused levels in the cervical spine has been studied. Schwab **et al**. reported that increased motion compensation occurred at segments immediately adjacent to a single-level fusion.^21^ Cunningham **et al**. showed that when an arthroplasty is performed adjacent to an arthrodesis, its ROM significantly increases when compared with the intact or single-level arthroplasty groups. He also demonstrated that there were changes in the adjacent-level centres of rotation after arthrodesis.^22^ Dimitriev **et al**. reported that the adjacent level intradiscal pressure was significantly higher in the arthrodesis group when compared to both the intact and disc replacement groups.^23^ Finally, KFS is a congenital condition with pre-existing degenerative changes throughout the cervical spine. The kinematics of the cervical spine in KFS, therefore, likely behave differently than other situations where there is a prior one- or even two-level fusion, but there are no biomechanical studies specific to this rare condition. These biomechanical factors may be the potential reason for arthroplasty failure in our patient and perhaps only patients with limited disease (type I according to Samartzis^14^) should be treated with multilevel arthroplasty.

We present a case in which an artificial cervical disc device failed to address the anatomic and biomechanical challenges of global previous fusion in a patient with KFS. The use of arthroplasty in an extensively stiff and fused neck is challenging and may lead to hardware failure. Although motion preservation is desirable in Klippel-Feil patients, the kinematics and biomechanics of their cervical spine may hinder arthroplasty. Each case should be individualised and fusion or hybrid constructs may represent more reasonable options, especially with patients with multiple fused segments.

## References

[1] Baaj AA, Uribe JS, Vale FL, Preul MC, Crawford NR (2009). History of cervical disc arthroplasty. Neurosurg Focus.

[2] Eck JC, Humphreys SC, Lim TH, Jeong ST, Kim JG, Hodges SD (2002). Biomechanical study on the effect of cervical spine fusion on adjacent-level intradiscal pressure and segmental motion. Spine (Phila Pa 1976).

[3] Goffin J, Casey A, Kehr P, Liebig K, Lind B, Logroscino C (2002). Preliminary clinical experience with the Bryan Cervical Disc Prosthesis. Neurosurgery.

[4] Robertson JT, Papadopoulos SM, Traynelis VC (2005). Assessment of adjacent-segment disease in patients treated with cervical fusion or arthroplasty: A prospective 2-year study. J Neurosurg Spine.

[5] Kim SW, Limson MA, Kim SB, Arbatin JJ, Chang KY, Park MS (2009). Comparison of radiographic changes after ACDF versus Bryan disc arthroplasty in single and bi-level cases. Eur Spine J.

[6] Denaro V, Papalia R, Denaro L, Di Martino A, Maffulli N (2009). Cervical spinal disc replacement. J Bone Joint Surg Br.

[7] Pickett GE, Sekhon LH, Sears WR, Duggal N (2006). Complications with cervical arthroplasty. J Neurosurg Spine.

[8] Goffin J, Van Calenbergh F, van Loon J, Casey A, Kehr P, Liebig K (2003). Intermediate follow-up after treatment of degenerative disc disease with the Bryan Cervical Disc Prosthesis: Single-level and bi-level. Spine (Phila Pa 1976).

[9] Yi S, Kim KN, Yang MS, Yang JW, Kim H, Ha Y (2010). Difference in occurrence of heterotopic ossification according to prosthesis type in the cervical artificial disc replacement. Spine (Phila Pa 1976).

[10] Tracy MR, Dormans JP, Kusumi K (2004). Klippel-Feil syndrome: Clinical features and current understanding of etiology. Clin Orthop Relat Res.

[11] Klimo P, Rao G, Brockmeyer D (2007). Congenital anomalies of the cervical spine. Neurosurg Clin N Am.

[12] Smoker WR, Khanna G (2008). Imaging the craniocervical junction. Childs Nerv Syst.

[13] Crockard HA, Stevens JM (1995). Craniovertebral junction anomalies in inherited disorders: Part of the syndrome or caused by the disorder?. Eur J Pediatr.

[14] Samartzis DD, Herman J, Lubicky JP, Shen FH (2006). Classification of congenitally fused cervical patterns in Klippel-Feil patients: Epidemiology and role in the development of cervical spine-related symptoms. Spine (Phila Pa 1976).

[15] Clarke RA, Catalan G, Diwan AD, Kearsley JH (1998). Heterogeneity in Klippel-Feil syndrome: A new classification. Pediatr Radiol.

[16] Leung CH, Ma WK, Poon WS (2007). Bryan artificial cervical disc arthroplasty in a patient with Klippel-Feil syndrome. Hong Kong Med J.

[17] Yi S, Kim SH, Shin HC, Kim KN, Yoon DH (2007). Cervical arthroplasty in a patient with Klippel-Feil syndrome. Acta Neurochir (Wien).

[18] Cardoso MJ, Rosner MK (2010). Multilevel cervical arthroplasty with artificial disc replacement. Neurosurg Focus.

[19] Cho BY, Lim J, Sim HB, Park J (2010). Biomechanical analysis of the range of motion after placement of a two-level cervical ProDisc-C versus hybrid construct. Spine (Phila Pa 1976).

[20] Barbagallo GM, Assietti R, Corbino L, Olindo G, Foti PV, Russo V (2009). Early results and review of the literature of a novel hybrid surgical technique combining cervical arthrodesis and disc arthroplasty for treating multilevel degenerative disc disease: Opposite or complementary techniques?. Eur Spine J.

[21] Schwab JS, Diangelo DJ, Foley KT (2006). Motion compensation associated with single-level cervical fusion: Where does the lost motion go?. Spine (Phila Pa 1976).

[22] Cunningham BW, Hu N, Zorn CM, McAfee PC (2010). Biomechanical comparison of single- and two-level cervical arthroplasty versus arthrodesis: Effect on adjacent-level spinal kinematics. Spine J.

[23] Dmitriev AE, Cunningham BW, Hu N, Sell G, Vigna F, McAfee PC (2005). Adjacent level intradiscal pressure and segmental kinematics following a cervical total disc arthroplasty: An in vitro human cadaveric model. Spine (Phila Pa 1976).

